# Dysuria, Urinary Retention, and Inguinal Pain as Manifestation of Sacral Bannwarth Syndrome

**DOI:** 10.1155/2015/185917

**Published:** 2015-11-17

**Authors:** Josef Finsterer, Johannes Dauth, Kurt Angel, Mateusz Markowicz

**Affiliations:** ^1^Krankenanstalt Rudolfstiftung, Postfach 20, 1180 Vienna, Austria; ^2^Private Office, Vienna, Austria; ^3^Urological Department, Krankenanstalt Rudolfstiftung, Vienna, Austria; ^4^Institute for Hygiene and Applied Immunology, Medical University of Vienna, Austria

## Abstract

Only few cases with sacral radiculitis due to infection with* Borrelia burgdorferi* leading to neurogenic urinary dysfunction have been reported. A 57-year-old male developed urethral pain and urinary retention, requiring permanent catheterization. Extensive urological investigations did not reveal a specific cause, which was why neurogenic bladder dysfunction was suspected. Neurologic exam revealed only mildly reduced tendon reflexes. Cerebral and spinal MRI were noninformative. CSF investigations, however, revealed pleocytosis, elevated protein, and antibodies against* Borrelia burgdorferi*. Intravenous ceftriaxone for three weeks resulted in immediate improvement of bladder dysfunction, with continuous decline of residual urine volume and continuous increase of spontaneous urine volume even after removal of the catheter and initiation of self-catheterization. Sacral radiculitis due to infection with* Borrelia burgdorferi* is a potential cause of detrusor areflexia and urethral, perineal, inguinal, and scrotal pain and may be misinterpreted as cystitis or urethritis. Ceftriaxone may result in progressive recovery of bladder dysfunction and pain. Neuroborreliosis may manifest exclusively as neurourological problem.

## 1. Introduction

Bannwarth syndrome is a polyradiculitis due to infection with the spirochete* Borrelia burgdorferi* sensu lato (further* Borrelia*) [1]. In the majority of the cases, Bannwarth syndrome affects the limbs [1]. Only in a few cases, radiculitis of the sacral radices has been reported [2–7]. Additionally,* Borrelia* infection can be causative for interstitial cystitis, another cause of* Borrelia*-associated bladder dysfunction [8, 9]. Cystitis can be also induced by experimental* Borrelia* infection in mice [10]. Here we present another case with urinary dysfunction due to sacral polyradiculitis from infection with* Borrelia burgdorferi*.

## 2. Case Report

The patient is a 57-year-old male, height 184 cm, weight 84 kg, with a history of headache and neck pain starting five days prior to admission to a urological department. One day after the initial symptoms he developed urethral pain and urinary retention at night. On the next day he became unable to void. Routine urine examination by the general practitioner was normal. Urologic examination at an ambulatory ward excluded prostatitis or prostate hypertrophy. Tamsulosin was prescribed and a transurethral bladder catheter was inserted. Despite these measures he developed increasing urethral pain and attended another ambulatory urological unit three days later. Though urine examination was normal again, levofloxacin was prescribed. At midday he developed fever of 39° and was admitted to the urological department at night (hospital day (hd) 1). Blood tests (hd1, hd2) revealed mildly elevated C-reactive protein of 11.8 mg/L and 13.4 mg/L, respectively (*n*, 0–5 mg/L), and elevated liver function parameters but were otherwise normal. Urine culture (hd2), PAP smear of the urethra (hd4), and blood culture (hd5) were all negative. Nevertheless, levofloxacin was continued for 8 days. Since he did not tolerate the catheter because of urethral pain, it was changed to a percutaneous cystostomy on hd4. Cystoscopy on hd9 did not show a mechanical, inflammatory, or neoplastic cause of bladder dysfunction. Urodynamic evaluation on hd9 revealed a first urge at 250 mL but detrusor areflexia at 420 mL. Tamsulosin was discontinued and replaced by bethanechol chloride. A neurogenic cause of urinary dysfunction was assumed and the patient was referred to the neurologist.

The patient had a previous history of increased ocular pressure, elevated gamma-glutamate-transpeptidase, and reported recurrent tick bites. He was a lazy voider for years. The family history was positive for mesenteric embolism (father), Parkinson syndrome (mother), hyponatremia and hypokalemia shortly before decease (mother), pulmonary embolism (brother), and deep venous thrombosis (son). Neurologic exam revealed mild bulb protrusion, sore neck muscles, and mildly reduced tendon reflexes exclusively. Nerve conduction studies of the peroneal and sural nerves were normal. MRI of the brain revealed only some lacunas in the basal ganglia. MRI of the cervical and thoracic spine showed multisegmental osteochondrosis, most prominent at C5/6, and spondylosis. MRI of the lumbar spine revealed hyperlordosis and mild protrusion of the discs L4/5 and L5/S1 and a sacral nerve root sleeve cyst (Tarlov cyst). Abdominal ultrasound revealed cholecystolithiasis and steatosis hepatis. Recommended lumbar puncture was not carried out. The patient was released on hd11 with the percutaneous cystostomy and bethanechol chloride.

**Figure 1:**
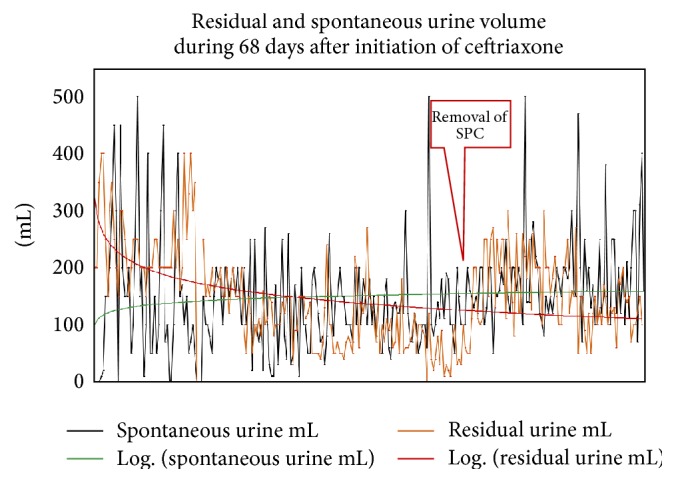
Course of residual urine volume and spontaneous urine volume after initiation of ceftriaxone during three weeks and during 6 weeks after discontinuation of ceftriaxone; SPC: suprapubic catheter.

Since dysuria persisted, and myalgias of the gluteal muscles, cold feet, inguinal, scrotal, and perineal pain had developed after dismissal, he was readmitted 7 days later. The neurologic exam was unchanged compared to the previous exam and lumbar puncture was carried out. CSF investigations revealed mild pleocytosis of 51/3 cells and increased total protein of 84 mg/dL (*n*, 20–40 mg/dL), which was why an intravenous therapy with ceftriaxone (2 g/d) for three weeks and acyclovir 1500 mg/d was initiated. Since all antibodies against common viruses and PCR for herpes types 1 and 2 and varicella zoster virus were negative in the CSF, the virostatic treatment was discontinued 13 days after initiation. Serum and CSF IgG antibodies against* Borrelia* were positive by ELISA (Medac Int.), >200 and 116 AU/mL, respectively. Immunoblot (Euroimmun) with serum revealed positive results, most intensively with the antigens VlsE, p39/BmpA, p25/OspC, p19, and p17. The IgG antibody index according to Reiber was 0.9 (*n*, <1.3). IgG in the CSF was elevated to 4.7 (*n*, <4.0) and CSF albumin was increased to 65.1 mg/dL (*n*, 35.0 mg/dL). CSF and blood culture were negative. PCR with CSF for* Borrelia* was negative. Three days after initiation of ceftriaxone the patient became almost free of pain and started to spontaneously contract the detrusor, to partially void, and to develop a feeling of filling and emptying of the bladder (Figure 1). Only during two days anuria recurred. He developed a normal urine stream and the residual urine volume continuously decreased (Figure 1).

After the second dismissal urethral pain had almost resolved but erection was painful. Residual urine volumes further declined (Figure 1). Six days after dismissal, however, urethral pain recurred and the patient experienced bladder pain and diarrhea. Urine examination was positive for leukocytes, protein, and hemoglobin. Prulifloxacin was prescribed by the general practitioner. Seven days after dismissal the patient was admitted to a tertiary center for urology during two days. Nerve conduction studies of the pudendal nerve and pudendal somatosensory-evoked potentials were normal. X-ray of the urethra suggested previous infections with* Neisseria gonorrhoeae* despite a negative history for such an infection. PAP smear of the urethra was normal again. After the third dismissal symptoms resolved and residual urine volumes further declined (Figure 1). Because urethral pain had recurred 5 days after the third dismissal, a third smear of the urethra was taken but it was normal again. Gram staining showed plenty of leukocytes, moderate epithelial cells, no bacteria, in particular* Neisseria gonorrhoeae*, but anaerobe* Staphylococcus epidermidis*. An anaerobe culture was negative. There was no growth of* Chlamydia trachomatis*, mycoplasmas, ureaplasmas,* Trichomonas vaginalis*, or yeasts. PCR for* Chlamydia trachomatis* was negative. A second urodynamic examination 18 days after the third dismissal was indicative of detrusor-sphincter dyssynergia, which is why bethanechol chloride was discontinued and tamsulosin was reestablished. After removal of the suprapubic catheter, 20 days after the third dismissal, the patient was introduced to intermittent self-catheterization. One day later he was free of pain and urine investigation was normal. However, residual urine volumes persisted at ~200 mL, which is why he continued self-catheterization since then.

## 3. Discussion

Besides characteristic meningoradiculoneuritis (Bannwarth syndrome), Lyme neuroborreliosis manifests with a variety of neurological abnormalities, such as encephalitis, dementia, or radiculomyelitis [1, 11]. A more rare manifestation is lower urinary tract dysfunction [3]. The urinary tract may be affected in two ways: first, as weakness of the detrusor due to affection of the innervating fibers by radiculitis, or, second, as detrusor weakness due to direct invasion of the spirochete into the bladder wall [3]. Both types of affection may lead to detrusor hyperreflexia, detrusor areflexia, or detrusor-sphincter dyssynergia [3]. In the majority of these cases, ceftriaxone is beneficial [12].

Sacral Bannwarth syndrome was diagnosed in the presented patient upon the history, clinical exam, CSF findings, and the favorable response to ceftriaxone. Cystitis due to* Borrelia* was excluded upon cystoscopy and upon the negative urine cultures. Experimental infection of hamsters showed that* Borrelia* have a marked tropism for the myocardium and the urinary tract tissues [13]. In 90% of the samples,* Borrelia* can be cultured from the bladder wall [13]. Persistent infection could be found in 78% of the urinary bladders [13]. Furthermore, experimentally infected mice showed lymphoid aggregates and vascular changes, such as increase in the number of vessels, thickening of the vessel wall, and perivascular infiltrates [10]. In 93% of these animals cystitis was found [10]. In another experimental study spirochetes could be isolated from the bladder wall in 94% of the mice [14].

Several arguments can be raised to support the notion that the infection of the CNS was indeed causative for bladder dysfunction in the presented patient. First, the patient had a history of tick bites. Second, CSF investigations showed mild pleocytosis, elevated CSF protein, presence of IgG antibodies against* Borrelia* in serum and CSF, and an intense reaction of serum IgG antibodies with immunodominant antigens of* Borrelia* in an immunoblot. Third, Bannwarth syndrome has been previously reported to cause bladder dysfunction [3]. In all these cases antibiotic treatment had a beneficial effect leading to partial or complete recovery. Fourth, there is broad evidence from the literature that experimentally infected animals show tropism for the infection of the lower urinary tract. In mice experimentally infected with* Borrelia*, spirochetes can be found by immune fluorescence stain in the urinary bladder wall but not in the urine [14]. Fifth, there was a beneficial effect of ceftriaxone with almost immediate recovery of the detrusor function after 14 d of anuria. The reasons why there was no complete recovery during the observational period remain speculative but possibly the infection had lasted already too long without antibiotic treatment or the follow-up period was too short to document complete recovery. Theoretically, infection with another agent could have been causative since ceftriaxone can be effective in infections other than* Borreliosis*. In the presented patient, urologists also suspected a subclinical preexisting damage of the detrusor muscle by chronic overextension or infection. Additionally, detrusor weakness due to a generalized metabolic defect can be also not completely excluded.

This case shows that sacral radiculitis due to infection with* Borrelia burgdorferi* is a potential cause of detrusor areflexia, that perineal, inguinal, and scrotal pain may be misinterpreted as cystitis or urethritis, that intolerance of a transurethral catheter due to urethral pain may be also attributable to radiculitis, and that ceftriaxone may result in continuous recovery of bladder dysfunction. Neuroborreliosis does not necessarily need to go along with neurological deficits but may manifest exclusively as neurourological problem.

## References

[1] Hansen K., Crone C., Kristoferitsch W. (2013). Lyme neuroborreliosis. *Handbook of Clinical Neurology*.

[2] Ackermann R., Gollmer E., Rehse-Küpper B. (1985). Progressive Borrelia encephalomyelitis. Chronic manifestation of erythema chronicum migrans disease of the nervous system. *Deutsche Medizinische Wochenschrift*.

[3] Chancellor M. B., McGinnis D. E., Shenot P. J., Kiilholma P., Hirsch I. H. (1993). Urinary dysfunction in Lyme disease. *Journal of Urology*.

[4] Chancellor M. B., Mcginnis D. E., Shenot P. J., Hirsch I. H., Kiilholma P. J. (1992). Lyme cystitis and neurogenic bladder dysfunction. *The Lancet*.

[5] Shamim E. A., Shamim S. A., Liss G., Nylen E., Pincus J. H., Yepes M. (2005). Constipation heralding neuroborreliosis: an atypical tale of 2 patients. *Archives of Neurology*.

[6] Chancellor M. B., Dato V. M., Yang J. Y. (1990). Lyme disease presenting as urinary retention. *Journal of Urology*.

[7] Dupuis M. J. M. (1988). Multiple neurologic manifestations of *Borrelia burgdorferi* infection. *Revue Neurologique*.

[8] Schwan T. G., MacDonald A. B. (1989). Interstitial cystitis and *Borrelia burgdorferi*. *Annals of Internal Medicine*.

[9] Haarala M., Kiiholma P., Nurmi M., Uksila J., Alanen A. (2000). The role of *Borrelia burgdorferi* in interstitial cystitis. *European Urology*.

[10] Czub S., Duray P. H., Thomas R. E., Schwan T. G. (1992). Cystitis induced by infection with the Lyme disease spirochete, *Borrelia burgdorferi*, in mice. *The American Journal of Pathology*.

[11] Halperin J. J. (2014). Nervous system Lyme disease. *Handbook of Clinical Neurology*.

[12] Kim M.-H., Kim W. C., Park D.-S. (2012). Neurogenic bladder in lyme disease. *International Neurourology Journal*.

[13] Goodman J. L., Jurkovich P., Kodner C., Johnson R. C. (1991). Persistent cardiac and urinary tract infections with *Borrelia burgdorferi* in experimentally infected syrian hamsters. *Journal of Clinical Microbiology*.

[14] Schwan T. G., Burgdorfer W., Schrumpf M. E., Karstens R. H. (1988). The urinary bladder, a consistent source of *Borrelia burgdorferi* in experimentally infected white-footed mice (*Peromyscus leucopus*). *Journal of Clinical Microbiology*.

